# Nebivolol And Quinapril Reduce P-Wave Duration And Dispersion In Hypertensive Patients

**Published:** 2009-05-15

**Authors:** Hasan Korkmaz, Orhan Onalan, Mehmet Akbulut, Yilmaz Ozbay

**Affiliations:** 1 Department of Cardiology, Elazig Education and Research Hospital, Elazig / Turkey; 2 Department of Cardiology, Gaziosmanpasa University. Tokat / Turkey; 3 Department of Cardiology, Firat University, Elazig / Turkey

**Keywords:** P-wave duration, Nebivolol, Quinapril, Hypertension

## Abstract

We aimed to investigate the effects of nebivolol and quinapril treatments on P-wave duration and dispersion in hypertensive patients. Hypertensive patients who were in sinus rhythm were assigned to the two treatment groups and received either 20 mg quinapril/day or 5 mg nebivolol/day. P-Wave dispersion (PWD) was measured at baseline and after four weeks of treatment and defined as the difference between the maximum (Pmax) and the minimum (Pmin) P-wave duration. The study group consisted of 54 patients (Mean age: 53 ±  9 years, 46% women) with 27 patients in each group. At 4-week follow up both treatment groups showed a significant reduction (p< 0.001) in systolic (SBP) and diastolic blood pressure (DBP). Heart rate (HR) reduction was significant in patients receiving nebivolol (P=0.001). Both groups showed a similar (P=0.413 for PWD, p=0.651 for Pmax) but significant reduction in PWD (nebivolol: -16± 14, P< 0.0001 and quinapril: -13± 11, P< 0.0001) and Pmax (nebivolol: -10± 11, P=0.001 and quinapril: -9± 11, P=0.001). A 2 (Time) x 2 (Group) mixed-model repeated-measures analysis of variance revealed that the main effect of Time was significant for Pmax (P=0.002) and PWD (P=0.008) after controlling for changes in SBP, DBP and HR. However, the main effect of Group and Time x Group interaction was not significant for both variables (All p values > 0.05). In conclusion, short-term treatment with nebivolol and quinapril produces a similar but significant reduction in Pmax and PWD in hypertensive patients. This effect is independent of blood pressure and heart rate changes.

## Introduction

The surface electrocardiogram (ECG) is the most common non-invasive method used for cardiovascular evaluations. Increased P-wave dispersion (PWD) and maximum P-wave duration (Pmax) are considered to be the non-invasive indicators of prolonged and heterogeneous atrial conduction  [1]. Accordingly, increased PWD and Pmax have shown to be useful non-invasive tools in prediction of the atrial fibrillation (AF) [1-4]. Hypertensive patients have an increased risk of AF which may be related to impaired homogeneity of atrial conduction [2]. Both angiotensin converting enzyme (ACE) inhibitors and beta blockers are widely used drugs for the treatment of hypertension. Renin angiotensin system (RAS) blockade by either ACE inhibitors or angiotensin II receptor blockers (ARBs) is associated with a reduced risk of AF in patients with hypertension or heart failure [5,6]. Beta-blockers reduces incidence of AF in patients with heart failure [7]. Nebivolol has a unique chemical structure and pharmacologic profile, which combines highly selective β 1- adrenergic receptor blockade with vasodilating action [8]. To date no study compared effect of beta-blockers and ACE inhibitors on P-wave duration and dispersion. Here we aimed to investigate the effect of nebivolol and quinapril on P-wave duration and dispersion in newly diagnosed hypertensive patients.

## Methods

The study included newly diagnosed hypertensive patients who were in sinus rhythm. Blood pressure measurements were done in the morning for the 3 consecutive days with an appropriate sized cuff mercury sphygmomanometer (ERKA, Bad Tolz, Germany). The patients were allowed to take a rest at least 10 minutes before each blood pressure measurement, without tea and coffee intake or smoking at least 30 minutes prior to the measurement. Patients who had a blood pressure value of ≥ 140/90 mm Hg and < 180/110 mmHg were included. We applied the following exclusion criteria: presence of (1) malignant or secondary hypertension; (2) ischemic or valvular heart disease; (3) heart failure; (4) renal dysfunction; (5) sustain atrial or ventricular arrhythmias; (6) antiarrhythmic treatment; (7) chronic obstructive lung disease or pulmonary hypertension; (8) neurological, oncological or rheumatological disease; (9) pregnancy; (10) a body mass index (BMI)  ≥ 35 kg/m^2^; (11) technically insufficient echocardiographic images; (12) left ventricular ejection fraction (LVEF) < 55%; (12) QRS duration ≥  120 millisecond (ms); (13) unrecognized P-wave in ≥  3 leads; (14) any  contraindication to beta-blockers or ACE inhibitors. Eligible patients were approached for study participation and informed consent was obtained from all participants. The recruitment of the patients was completed prospectively in 6 months between December 1st, 2005 and May 31st, 2006.

Electrocardiograms were obtained using a 3-channel standard 12-lead synchronous electrocardiography apparatus (Cardiofax GEM-9020 K, Nihon Kohden, Japan) with a rate of 50 mm/seconds and amplitude of 10 mV including at least 3 QRS complexes for each derivation. During recordings, the patients were allowed to breathe freely, but not to speak. The P-wave durations for each derivation was manually analyzed through a x10 magnifying glass. The beginning of P-wave was considered as intersection point of isoelectric line with the P-wave while the end point was the intersection of isoelectric line with the last point of P-wave. The difference between the longest and shortest P-waves was defined as PWD. All measurements were done by the same cardiologist who was blinded to clinical characteristics of the participants.

A transthoracic echocardiography was performed in all patients using a 3.2 MHz probe (Acuson Sequoia 512, Siemens, Germany). Parasternal long- and short-axis, apical four chamber and two chamber views were obtained, and left ventricular systolic and diastolic functions were evaluated. Left atrium diameter, posterior wall thickness (PWT), interventricular septal thickness (IVST), left ventricular end-systolic diameter (LVESD) and left ventricular end-diastolic diameter (LVEDD) were measured for each patient. The left ventricular muscle mass (LVM) was calculated with the Devereux formula [9]; LVM= 0.8 {1.04(IVST + LVESD  + PWT)^3^ - (LVEDD)^3^} + 0.6. The LVM index was calculated as LVM divided by body surface area (BSA). Left ventricular hypertrophy was considered present when the LVM index was > 125g/m^2^ in men, and > 110g/m^2^ in women [10].

The recruited patients were assigned to the two treatment groups in a sequential order and received either 20 mg quinapril/day or 5 mg nebivolol/day treatment. A brochure explaining life style changes (i.e. reducing weight and salt intake) was given to all patients. Lipid profile and renal function tests were performed for each patient, including the measurement of height and weight. BMI was calculated as weight in kilograms divided by height in square meters. The patients who had a BMI ≥  30 kg/m^2^ were defined as obese. All patients were reevaluated at 4-weeks follow up. No other antihypertensive drugs or any other drugs with effects on cardiovascular system were allowed during the study period.  The study was conducted in accordance to the Helsinki Declaration with the permission of the local ethical board.

## Statistical Analysis

Continuous data were expressed as the mean ±  standard deviation; categorical data were expressed as numbers with percentages. The Shapiro-Wilk test was used to assess the normality of the continuous data. Differences in continuous data were analyzed by using the Students' t or Mann-Whitney U test in two independent groups. Categorical data were compared by using the Chi-square test or Fisher's Exact test in two independent groups. T or Wilcoxon test for paired samples was used to assess changes in the variables over time within each group. A 2 (Time) x 2 (Group) mixed-model repeated-measures analysis of variance (ANOVA) was performed to compare between-group differences .Changes in P-wave parameters were adjusted for the changes in blood pressure and heart rate in this ANOVA model. A two tailed p value < 0.05 was considered statistically significant. All statistical analyses were performed using the SPSS (statistical Package for the Social Sciences) statistical software package (Version 12: SPSS Inc, Chicago, IL, USA).

## Results

A total of 62 consecutive patients, 31 in each group, were recruited. Two patients who didn't comply with the treatment regimen (1 in quinapril group, 1 in nebivolol group), and three patients who didn't attend the control visit at week 4 (2 in quinapril group, 1 in nebivolol group) and three patients who failed to follow the instructions (1 in quinapril group, 2 in nebivolol group) were excluded. Thus, the study group consisted of 54 patients (Mean age: 53 ±  9 years, 46% women) with 27 patients in each group. The baseline characteristics of the two study groups were comparable (Table 1). Mean blood pressure, heart rate, Pmax, minimum P-wave duration (Pmin) and PWD changes from baseline to week 4 (delta) in each group are shown in Table 2 and Figure. At 4-week follow up both treatment groups showed a significant reduction in systolic (SBP) and diastolic blood pressure (DBP). The delta DBP was significantly higher in the nebivolol group than in the quinapril group (-13± 9 vs. -8± 5, p=0.037) but delta SBP was similar between groups (-29± 11 vs. -24± 11, p=0.079, Table 3). Heart rate (HR) reduction was significant in the nebivolol treated patients (P=0.001) however, the difference in delta HR was not significant between two treatment groups (-7± 9 vs. -3± 14, P=0.112).

Both groups showed a similar but significant reduction in PWD and Pmax (Table 3). The delta PWD was -16± 14 ms (P<0.0001) and -13± 11ms (P<0.0001) in the nebivolol and quinapril group, respectively. Corresponding figure for Pmax was -10± 11 ms (P=0.001) and -9± 11 ms (P=0.001), respectively. The difference in delta PWD and delta Pmax were not statistically significant between two groups (P=0.413 and p=0.651, respectively).

A 2 (Time) x 2 (Group) mixed-model repeated ANOVA revealed that the main effect of Time was still significant for Pmax (P=0.002) and PWD (P=0.008) after controlling for delta SBP, delta DBP and delta HR (Table 4). However, again, the main effect of Group and Time x Group interaction was not significant for both variables (All p values >0.05).

During the study period, cough occurred only in one patient in the quinapril group. In the nebivolol group, one patient experienced headache, and one patient experienced weakness. No other clinical side effect or laboratory abnormality observed during the study period.

## Discussion

To our knowledge, this is the first study to compare the effect of beta- blockers and ACE inhibitors on the P-wave duration and dispersion. We showed that nebivolol and quinapril treatment produce a similar but significant reduction in Pmax and PWD in patients with newly diagnosed hypertension. Moreover, this effect was independent of blood pressure and heart rate changes.

Increased Pmax and PWD are believed to reflect heterogeneous and prolonged atrial conduction [1]. Independent of age, Pmax and PWD are predictors for the onset of AF in patients with hypertension [11]. Hypertension related changes in the left atrium including altered left atrial electrophysiology, increased left atrial pressure, stretch, dilatation and fibrosis are associated with development of AF [12,13]. Atrial electrophysiological changes can occur early in hypertension and may even precede left atrial enlargement [14]. Prolongation of atrial conduction times and shortening of the atrial effective refractory period (AERP) with loss of its rate dependence are the most characteristic features of atrial electrical remodeling [15]. Patients at very early stages of hypertension have demonstrable evidence of prolonged atrial conduction by P-wave signal-averaged ECG [14].

The density of angiotensin (Ang)-II receptors in the atria is generally greater than in the ventricles [16] and Ang-II may contribute  directly to atrial electrical remodeling [13,17]. Experimentally, the inhibition of endogenous Ang-II with candesartan and captopril prevented AERP shortening during rapid atrial pacing in dogs [17]. Stimulation of Ang II type 1 receptors promotes fibroblast proliferation and collagen synthesis and thereby atrial fibrosis [13,18]. Atrial fibrosis may cause heterogeneity in atrial conduction and has been suggested to play a role in occurrence of AF [12,13]. Moreover, P-wave duration tends to increase as atrial fibrosis increases [19]. ACE inhibitors reduce the Ang-II dependant activation of several intracellular signaling pathways mediating cell growth, proliferation, fibrosis and inflammation [13,18]. Thus, modification of atrial electrical and/or structural remodeling with quinapril is probably one of the main mechanisms for Pmax and PWD reduction observed in this study. Activation of the sympathetic nervous system may also contribute to prolongation and heterogeneity of atrial conduction [20,21]. RAS inhibition can modulate sympathetic nervous system activity [18]. Inflammation is increasingly recognized as a central feature in the pathogenesis of AF [22]. Thus, another potential mechanism in this setting may be associated with anti-inflammatory action of RAS inhibition [18,23]. Blocking the RAS can therefore offer an incremental benefit beyond its blood pressure lowering effect.  Consequently, a recent meta-analysis of 11 trials, revealed that RAS inhibition with ACE inhibitors or ARBs reduces incidence of AF in a wide range of diseases including hypertension, heart failure, myocardial infarction, and after electrical cardioversion [5].

There are several possible direct or indirect mechanisms by which nebivolol might reduce the prolongation of the maximum duration and dispersion of the P-wave. Beta-blockers lengthen duration of diastole, enhance ventricular filling, decrease atrial pressure, and dilatation and thereby may improve stretch related arrhythmogenic mechanisms [11,24,25]. Increased sympathetic drive has been shown to induce atrial tachyarrhythmias [26] and beta blockers prevented AF recurrences in patients with hypertension [27,28]. Thus, another potential explanation may be the attenuation of the actions of the sympathetic nervous system on atrial electrical physiology. Hypertension may also induce atrial ischemia which further contributes to the heterogeneous and prolonged atrial conduction. Beta-blockers may reduce atrial ischaemia and fibrosis in those with underlying ischemic heart disease [29,30]. Nebivolol may promote nitric oxide (NO) bioactivity and vasodilatation by inhibiting oxidative stress and inflammation [8]. Moreover, nebivolol significantly reduced coronary artery smooth muscle cell growth [31,32]. Combination of improved NO bioactivity, reduced oxidative stress, inflammation and inhibition of smooth muscle cell growth with nebivolol treatment may improve ischemia induced prolongation of atrial conduction. Consistent with this data, beta-blockers have been shown to be effective in adrenergically mediated AF [33], in prevention of postoperative AF [29,30] and in reducing relapse rates of AF following cardioversion [28]. Recently, in a meta-analysis of 7 trials, including 11952 heart failure patients, beta blockers significantly reduced incidence of AF [7].

In conclusion, antihypertensive treatment with nebivolol and quinapril produces a similar but significant reduction in PWD and Pmax. Our results also suggest that reduction of Pmax and PWD cannot be solely attributed to a better blood pressure control since effect of both quinapril and nebivolol on Pmax and PWD was independent of blood pressure and heart rate changes. Further studies are required to assess clinical utility of Pmax and PWD reduction after the administration of anti-hypertensive treatment for the prevention of AF.

Several limitations should be considered. This study is subject to the limitations inherent to a nonrandomized study. A second limitation of the study is the small sample size. P-wave measurements were performed manually on the standard paper ECG. A digitized measurement method for P-Wave parameters would be more accurate.

## Figures and Tables

**Table 1:**
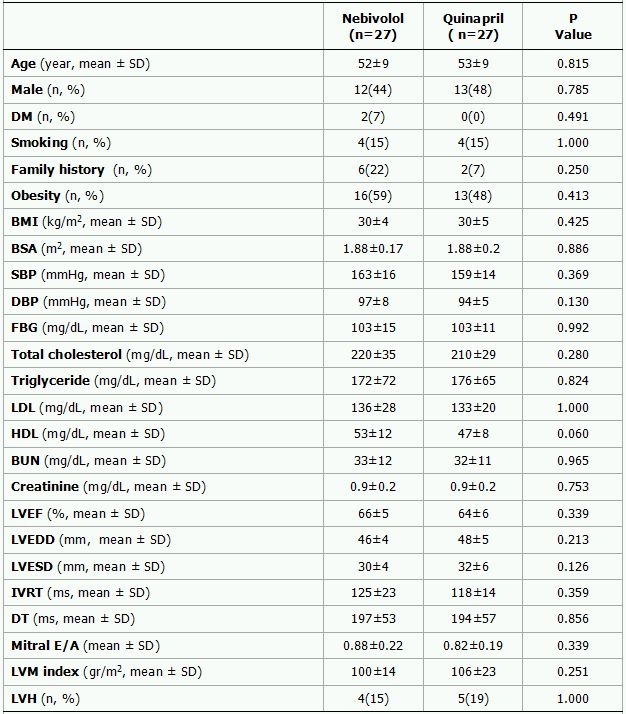
Baseline characteristics of the treatment groups

SD represent standard deviation; DM, diabetes mellitus; BMI, body mass index; BSA, body surface area; SBP, systolic blood pressure; DBP, diastolic blood pressure; FBG, fasting blood glucose; LVEF, left ventricular ejection fraction; LVEDD, left ventricular end-diastolic diameter; LVESD, left ventricular end-systolic diameter; IVRT, isovolumic relaxation time; DT, deceleration time; ms, millisecond; mm, millimeter; LVM, left ventricular mass; LVH, left ventricular hypertrophy

**Table 2:**
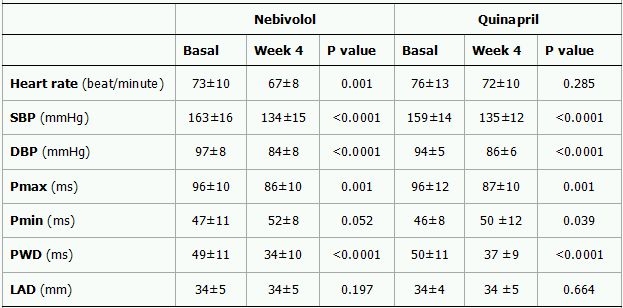
Pre- and post-treatment values of blood pressure, heart rate, P-wave parameters and left atrial diameter in the nebivolol and quinapril groups.

Data are expressed as mean ±  standard deviation. SBP represent systolic blood pressure; DBP, diastolic blood pressure; PMax, maximum P-wave; PMin, minimum P-wave; PWD, P-wave dispersion.

**Table 3:**
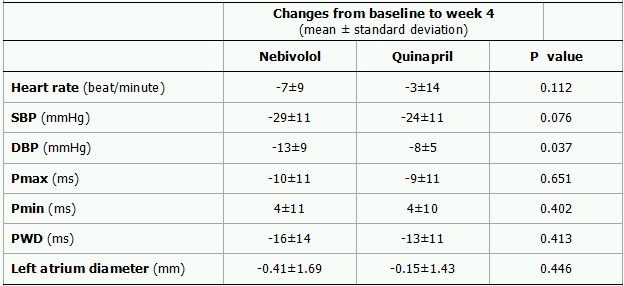
Changes in blood pressure, heart rate and P-wave parameters in the nebivolol and quinapril groups

SBP represent systolic blood pressure; DBP, diastolic blood pressure; Pmax, maximum P-wave; Pmin, minimum P-wave; PWD, P-wave dispersion; ms, millisecond; mm, millimeter.

**Table 4:**
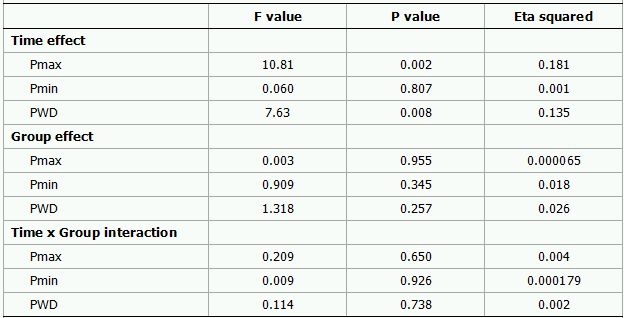
Main effect of Time, Group and Time x Group interaction on P-wave parametres

Analysis was based on a 2 (Time) x 2 (Group) mixed-model repeated-measures analysis of variance. The results were adjusted for the changes in blood pressure and heart rate. Pmax represent maximum P-wave; Pmin, minimum P-wave; PWD, P-wave dispersion.
